# TIGAR deficiency sensitizes angiotensin‐II‐induced renal fibrosis and glomerular injury

**DOI:** 10.14814/phy2.15234

**Published:** 2022-04-20

**Authors:** Xiaochen He, Aubrey C. Cantrell, Quinesha A. Williams, Jian‐Xiong Chen, Heng Zeng

**Affiliations:** ^1^ Department of Pharmacology and Toxicology University of Mississippi Medical Center School of Medicine Jackson Mississippi USA

**Keywords:** angiotensin II, fibrosis, glomerular injury, renal glycolysis, TIGAR

## Abstract

Angiotensin II (Ang‐II) is one of the major contributors to the progression of renal fibrosis, inflammation, glomerular injury, and chronic kidney disease. Emerging evidence suggests that renal glycolysis plays an important role in renal fibrosis and injury. TP53‐induced glycolysis and apoptosis regulator (TIGAR) has been shown to regulate glycolysis. In the present study, we investigated the role of TIGAR in renal glycolysis, fibrosis, and glomerular injury during Ang‐II‐induced hypertension. Wild‐type (WT) and TIGAR knockout (KO) mice were infused with Ang‐II (1 µg/kg/min) via mini‐pumps for 4 weeks. The mean arterial pressure was similar between the WT and TIGAR KO mice, associated with a comparable increase in plasma creatinine level. Ang‐II infusion resulted in a significant increase in renal interstitial fibrosis and more mesangial expansion and collapsed glomerular structure in the TIGAR KO mice. These were associated with elevated expression of hypoxia‐inducible factor‐1 alpha, glycolytic enzymes, and transforming growth factor beta 1 in the TIGAR KO mice after Ang‐II infusion when compared to that of the WT mice. The coupled‐enzyme method revealed that PFK‐1 activity was similarly increased in WT and TIGAR KO mice after Ang‐II infusion. Our present study suggests that TIGAR is involved in Ang‐II‐induced renal fibrosis and glomerular injury, although it has little effect on blood pressure and renal function. Knockout of TIGAR sensitizes Ang‐II‐induced renal fibrosis and injury. This study provides new insights into the role of TIGAR in renal metabolism and pathological remodeling during Ang‐II‐induced hypertension.

## Graphical abstract

Knockout of TIGAR sensitizes Ang II‐mediated HIF‐1α and TGF‐β expression in the mouse kidney. Knockout of TIGAR enhances Ang‐II‐induced increase in renal interstitial fibrosis as well as more mesangial expansion and collapsed capillaries. This study provides new insights into the role of TIGAR in renal metabolism and pathological remodeling during Ang‐II‐induced hypertension.

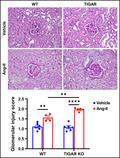

## 1 INTRODUCTION

Hypertension is closely associated with chronic kidney disease (CKD) (Judd & Calhoun, 2015; Pugh et al., 2019). The mechanisms of hypertension in CKD include volume overload, neuronal/hormonal alterations, salt retention, and endothelial dysfunction (Ku et al., 2019), in which angiotensin II (Ang‐II), the main player of the renin–angiotensin system (RAS), is one of the major contributors to the progression of renal fibrosis, inflammation, glomerular injury, and CKD (Haase, 2011; Mezzano et al., 2001).

TP53‐induced glycolysis and apoptosis regulator (TIGAR) plays an important role in glycolysis in which phosphofructokinase 1 (PFK‐1) is a rate‐limiting enzyme in this process. PFK‐1 activity is regulated by 6‐phosphofructo‐2‐kinase/fructose‐2, 6‐bisphosphatase isoform 3 (PFKFB3) which produces fructose 2,6‐bisphosphate (F2,6‐BP), a potent allosteric activator of PFK‐1 (De Bock et al., 2013; Schoors et al., 2014; Xu et al., 2014), whereas TIGAR decreases the level of F2,6‐BP and inhibits glycolysis (Bensaad et al., 2006; Green & Chipuk, 2006). Glucose metabolism in kidney plays a critical role in renal function not only because glucose is crucial in electrolyte transport, but also a key fuel for generating ATP, particularly in glycolysis (Ross et al., 1986). Emerging evidence suggests that aerobic glycolysis was upregulated in the activated renal interstitial fibroblasts, whereas inhibition of aerobic glycolysis attenuated renal fibrosis (Ding et al., 2017). Ang‐II has been shown to induce mitochondrial dysfunction and alter cardiac substrate use and the metabolomic profile in cardiac hypertrophy (Doughan et al., 2008; Mervaala et al., 2010). Ang‐II also induced glycolysis in endothelial cells and endothelial‐to‐mesenchymal transition (Gao et al., 2020). A recent study by Kim et al demonstrated that TIGAR was upregulated during renal ischemia–reperfusion injury (IRI), associated with impaired glycolysis, renal functional, and histological damage, whereas knockdown of TIGAR increased PFK‐1 activity and ATP, along with improved renal function and less tubular injury in the severe ischemic kidneys (Kim et al., 2015). Our recent study revealed that TIGAR deficiency protected the heart from Ang‐II induced hypertrophy, but did not affect cardiac function or fibrosis (He, et al, unpublished data). Our previous study demonstrated that Sirtuin 3 deficiency that upregulated TIGAR in cardiomyocytes (Li et al., 2021) also sensitized Ang‐II‐induced renal fibrosis via promoting pericyte‐fibroblast transition, iron overload, and reactive oxygen species (ROS) (Feng et al., 2020). These studies suggest that TIGAR is involved in the Ang‐II‐induced pathological effects. However, the exact mechanism by which TIGAR regulates renal function and fibrosis has never been investigated.

The present study examined whether TIGAR‐mediated glycolysis played a role in chronic Ang‐II‐induced renal dysfunction, glomerular injury, and fibrosis by using the TIGAR knockout (TIGAR KO) mice. The results demonstrate that knockout of TIGAR increased the expression of PFK‐1 and that chronic infusion of Ang‐II increased the level of HIF‐1α, PFKFB3, and TGF‐β1 in the TIGAR KO mice, associated with more renal interstitial fibrosis and glomerular injury than the WT mice. Our present data suggest a fundamental role of TIGAR in renal glycolysis and the development of fibrosis and glomerular injury in Ang‐II‐induced hypertension.

## 2 MATERIALS AND METHODS

### 2.1 Mice

Male C57BL/6J mice were purchased from The Jackson Laboratory and were used as wild‐type (WT) controls. Male TIGAR deficient (TIGAR KO) mice on the C57BL/6 background were kindly gifted by Dr. Jeffrey Pessin at the Albert Einstein College of Medicine and maintained in the Laboratory Animal Facilities at the University of Mississippi Medical Center (UMMC). All animals were fed laboratory standard chow and water and housed in individually ventilated cages. All protocols were approved by the Institutional Animal Care and Use Committee at UMMC (Protocol ID: 1564) and were in compliance with the National Institutes of Health Guide for the Care and Use of Laboratory Animals (NIH Pub. No. 85–23, Revised 1996).

### 2.2 Infusion of Ang‐II and measurement of blood pressure

WT and TIGAR KO mice (4 months old) were trained for blood pressure measurement by the tail‐cuff method (CODA Noninvasive Blood Pressure System, Kent Scientific, Torrington, CT) without anesthesia. One group of mice of each strain was infused with 1 µg/kg/min of Ang II for 4 weeks via subcutaneously implanted (midscapular region) Alzet mini osmotic pumps (*n* = 9/group). Pumps were implanted under isofluorane anesthesia. A control group was implanted with sterile saline‐filled pumps. After blood pressure measurements, body weights were recorded, and the animals were euthanized. The kidneys were rapidly excised, weighed, and snap‐frozen in liquid N_2_ for further analysis.

### 2.3 Plasma creatinine assay

Plasma creatinine level was measured by using a LabAssay^TM^ Creatinine assay kit (Fujifilm Wako Chemicals) according to the manufacturer's instructions. Briefly, plasma samples were deproteinized (sodium Tungstate and phosphoric acid) and centrifuged at 600 g for 10 min at room temperature. One hundred microliters of supernatant was then mixed with 50 µl of picric acid (22 mM) and 50 µl of NaOH (0.75 M) and incubated at 25–30°C for 20 min. The absorbance at 520 nm was then read within 30 min by a Bio‐Rad xMark microplate spectrophotometer (Bio‐Rad) and plotted against a standard curve. Data are expressed as mg/dl.

### 2.4 Phosphofructokinase activity assay

Tissue PFK‐1 activity was determined as previously described (Deng et al., 2008). Briefly, samples of tissues were weighted and homogenized in lysis buffer followed by sonication and centrifugation. The reaction was performed using 4 µg of total protein in a 96‐well plate containing 80 µl of the reaction buffer (50 mM Tris–HCl, pH 7.5, 5 mM MgCl_2_, 5 mM ATP (Sigma #A6419), 0.2 mM NADH, 100 mM KCl, 5 mM Na_2_HPO_4_, 0.1 mM AMP (Sigma #A2252), 5 mM fructose‐6‐phosphate, 5 U/ml triosephosphate isomerase, 1 U/ml aldolase, and 10 U/ml glycerol‐3‐phosphate dehydrogenase. The absorbance at 340 nm was read at 37°C every 30 s for a period of 30 min in a Bio‐Rad xMark microplate spectrophotometer. Data are expressed as the change in absorbance at 340 nm/min/mg of protein.

### 2.5 Histology

Kidneys were fixed with 10% neutral buffered formalin, processed, embedded in paraffin, and sectioned at 5‐μm thickness. Picrosirius red staining was used to evaluate the degree of interstitial fibrosis. Five to ten fields were randomly taken by using a Nikon Eclipse 80i microscope (Nikon Instruments) and analyzed by ImageJ software for each mouse, and the fibrotic fraction was calculated as the ratio of Picrosirius red‐stained area to total tissue area in the field.

Renal sections were stained with periodic acid‐Schiff (PAS) reagents to examine the glomerular injury and scored semiquantitatively in a blinded fashion (Sun et al., 2013; Uil et al., 2018; Zhao et al., 2020). Thirty to thiry five glomeruli/mice were scored for the presence of mesangial matrix expansion and loss of glomerular structure on a scale of 0–4, with 0 representing a normal glomerulus, 1 representing 1%–25%, 2 representing 26%–50%, 3 representing 51%–75%, and 4 representing >75% of mesangial expansion and loss of glomerular structure in the glomerular tuft.

### 2.6 Immunoblot analysis

Protein extractions from kidney samples were prepared in lysis buffer with protease/phosphatase inhibitor cocktail (A32961, Thermo Fisher Scientific). Lysates were separated by SDS‐–PAGE under reducing conditions, transferred to a PVDF membrane, and analyzed by immunoblotting. The PVDF membranes were probed with primary antibodies of HIF‐1α, PFKFB3, PFK1, and TGF‐β1 (Table S1). The membranes were then washed and incubated with an anti‐rabbit (31460) or anti‐mouse (31430) secondary antibody conjugated with horseradish peroxidase (1:10000, Thermo Fisher Scientific). Loading control were probed with β‐actin and GAPDH antibodies. Densitometries were analyzed in the Image Lab software 6.0 (Bio‐Rad).

### 2.7 Statistical analysis

Data are presented as mean ± SEM. The assumptions of normality in both comparison groups were determined by normality and long‐normality test. Statistical significance was determined by using Student's *t*‐test (two‐tailed) between the means of two groups, or two‐way ANOVA followed by Tukey's post hoc test for multiple comparisons in GraphPad Prism 8 software. *p* < 0.05 was considered statistically significant.

## 3 RESULTS

### 3.1 TIGAR deficiency does not affect renal function in Ang‐II‐induced hypertension

**FIGURE 1:**
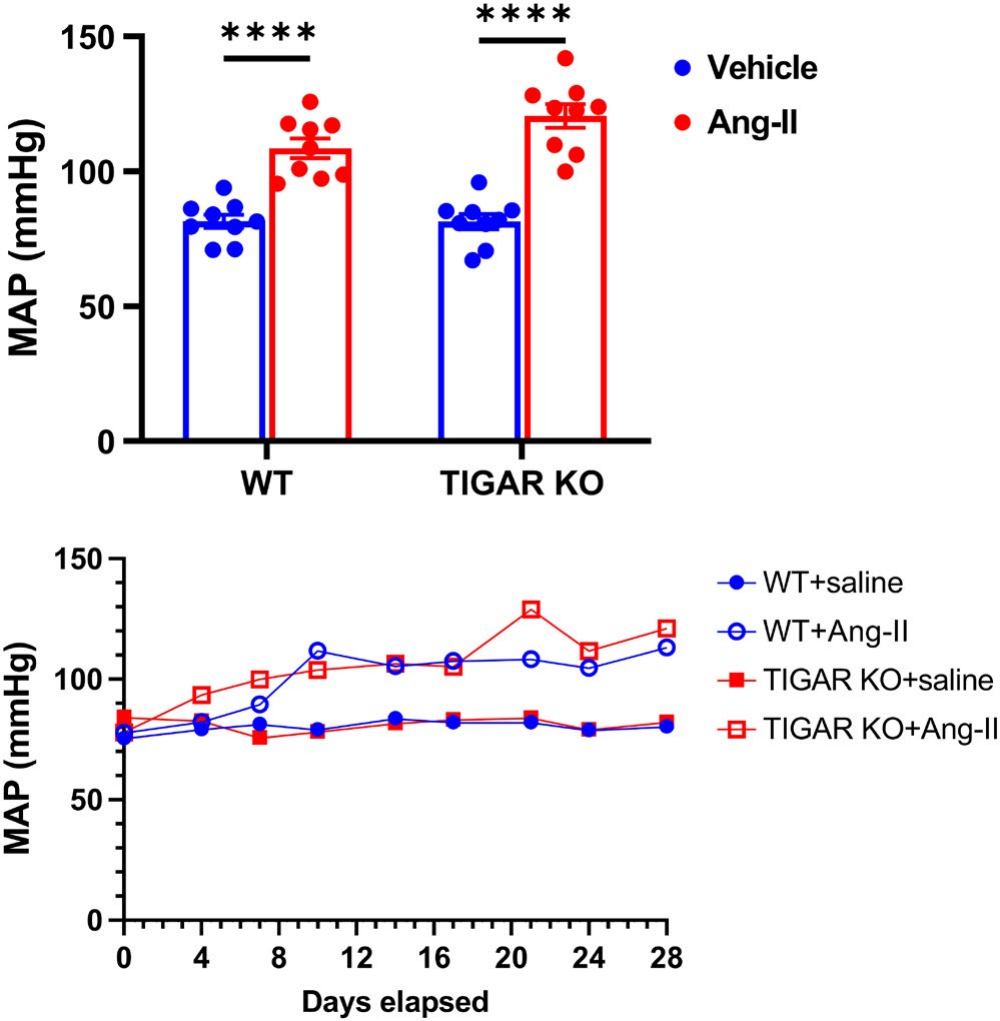
Effect of TIGAR deficiency on blood pressure in Ang‐II‐induced hypertension. Mean artery blood pressure (MAP) after 4 weeks of Ang‐II infusion was measured by the tail‐cuff method. *n* = 9. ****p < 0.0001

**FIGURE 2:**
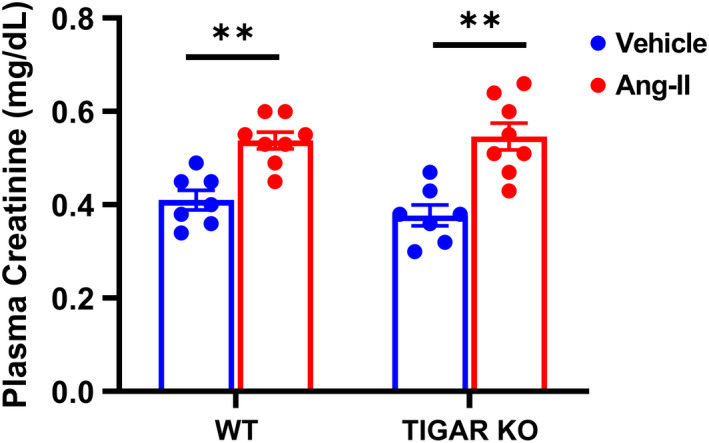
Effect of TIGAR deficiency on renal function in Ang‐II‐induced hypertension. Plasma creatinine level was measured by using the Jaffé method available as a LabAssay^TM^ Creatinine assay kit (Fujifilm Wako Chemicals, VA, USA). *n* = 7–8. **p < 0.01

To investigate the role of TIGAR in renal function during Ang‐II‐induced hypertension, WT and TIGAR KO mice were infused with Ang‐II (1 µg/kg/min) for 4 weeks. Ang‐II induced a significant increase in mean arterial pressure in both WT and TIGAR KO mice when compared to the corresponding vehicle controls, but there was no significant difference between the WT and TIGAR KO mice during 4 weeks of Ang‐II infusion (Figure 1). Plasma creatinine levels were also significantly increased in both WT and TIGAR KO mice after Ang‐II infusion, but there was no significant difference between the two groups (Figure 2), indicating the renal function was similarly impaired in both groups.

### 3.2 Knockout of TIGAR exacerbates Ang‐II‐induced renal fibrosis and injury

**FIGURE 3:**
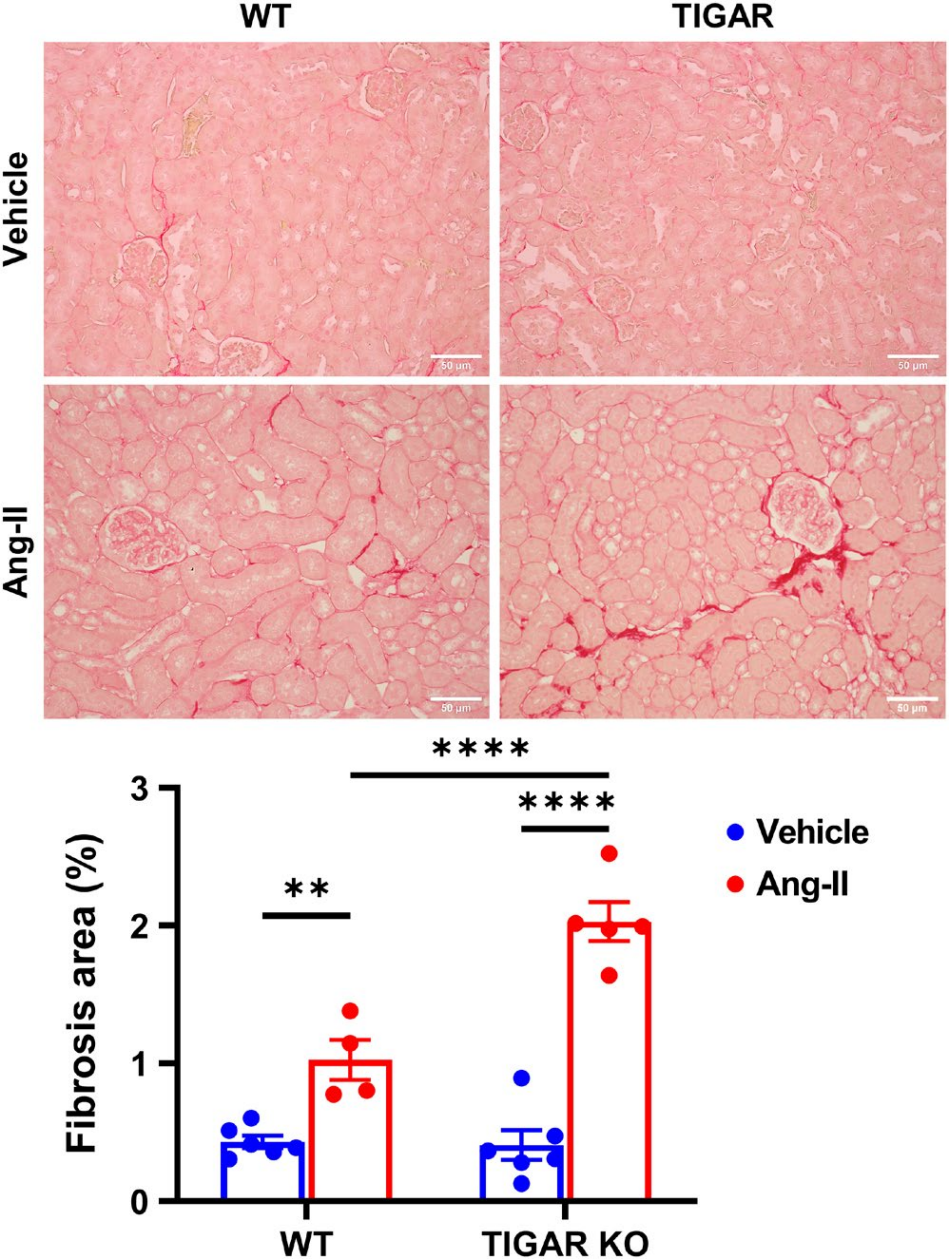
Knockout of TIGAR exacerbates Ang‐II‐induced renal fibrosis. The representative images of Picrosirius red‐stained paraffin‐embedded kidney sections (upper panel) showing interstitial fibrosis and quantification of fibrosis fraction (lower panel) in the indicated groups. Bar =50 μm. *n* = 4–6. **p < 0.01, ****p < 0.0001

**FIGURE 4:**
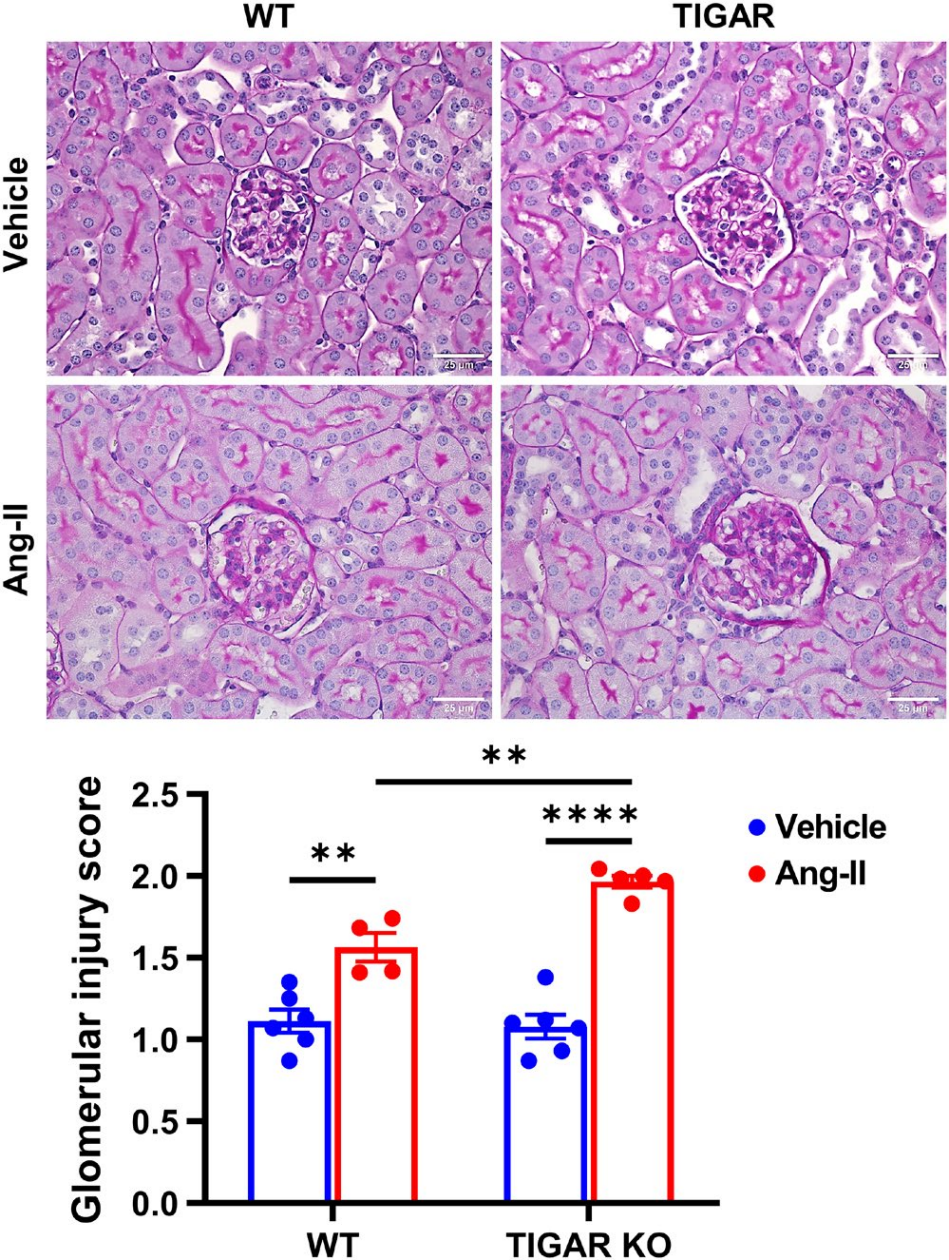
Knockout of TIGAR exacerbates Ang‐II‐induced glomerular injury fibrosis. The representative images of PAS‐stained paraffin‐embedded kidney sections (upper panel) showing mesangial expansion and collapsed structure in the glomeruli after infusion with Ang‐II and semi quantification of glomerular injury score (lower panel) in the indicated groups. Bar =25 μm. *n* = 4–6. **p < 0.01, ****p < 0.0001

Ang‐II‐induced interstitial fibrosis was assessed by Picrosirius red staining. Ang‐II only induced a mild increase in renal interstitial fibrosis in the WT mice when compared to its vehicle control (Figure 3). In contrast, Ang‐II induced a more robust increase in renal interstitial fibrosis in the TIGAR KO mice than its control, which was also significantly higher than that of the WT mice (Figure 3). Similar, WT mice infusion with Ang‐II induced a mild renal injury and the glomerular injury score, as manifested by mesangial expansion and collapsed glomerular structure (Figure 4). However, TIGAR KO mice exhibited more mesangial expansion and collapsed structure of glomerular than the WT mice, indicating that Ang‐II induced more severe glomerular injury in TIGAR KO than the WT mice.

### 3.3 Knockout of TIGAR augments glycolytic enzymes, hypoxic, and pro‐fibrotic signaling

**FIGURE 5:**
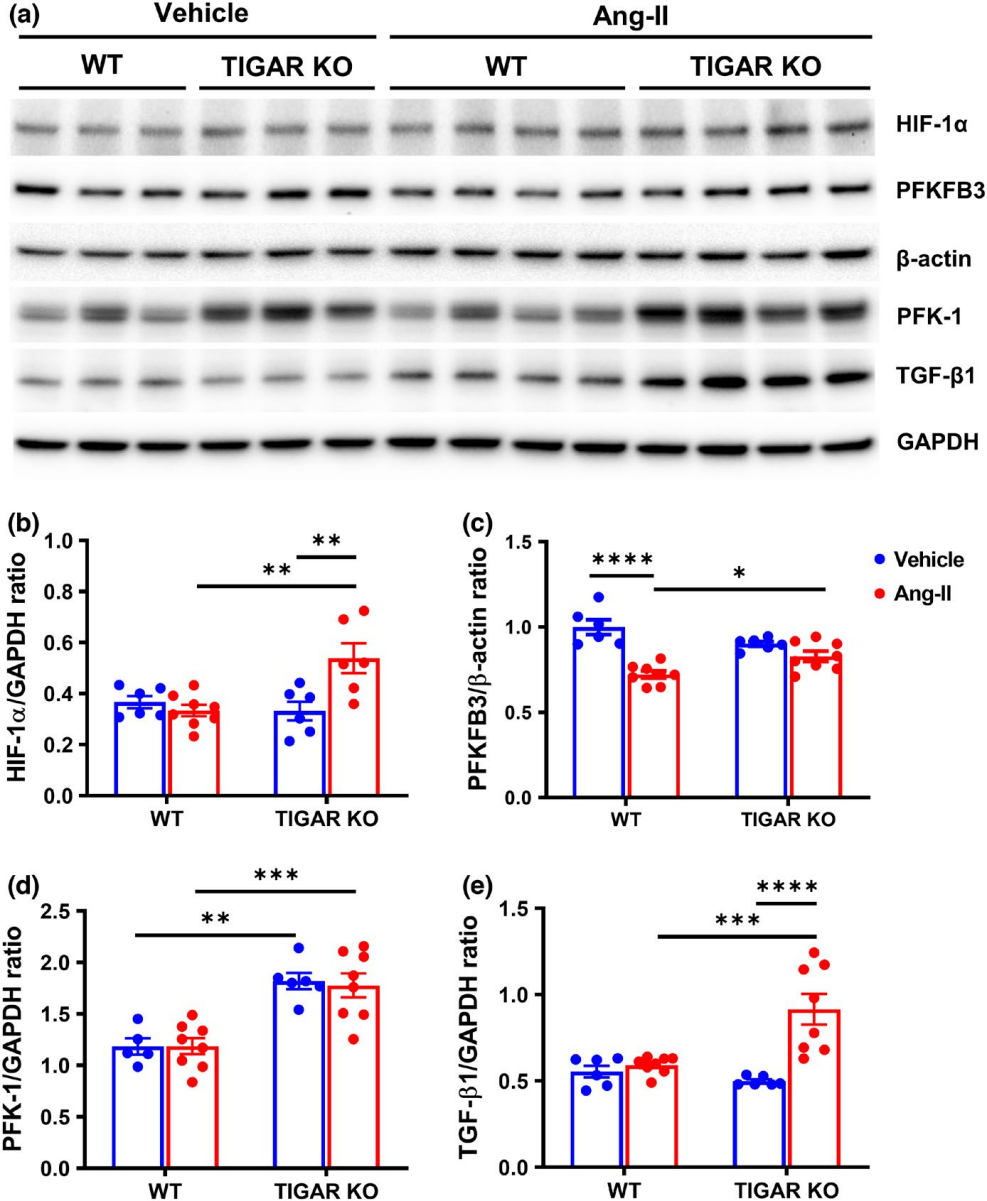
TIGAR deficiency increases glycolytic enzyme and pro‐fibrotic factor in Ang‐II‐induced hypertension. (a–e), Representative immunoblots and quantitative analysis of HIF‐1α, PFKFB3, PFK‐1, TGF‐β1, and corresponding GAPDH or β‐actin in the indicated groups. The expression of HIF‐1α and TGF‐β1 was significantly increased in the Ang‐II infused TIGAR KO mice, associated with elevated level of PFKFB3 and PFK‐1. *n* = 5–8, *p < 0.05, **p < 0.01, ***p < 0.001, ****p < 0.0001

**FIGURE 6:**
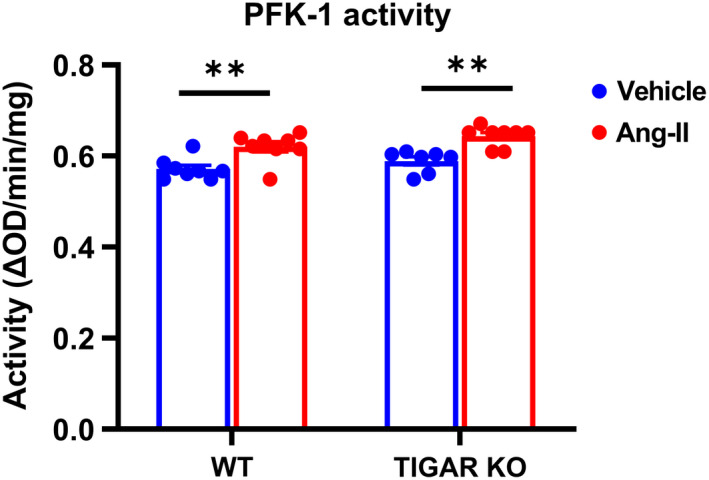
Ang‐II increases PFK‐1 activity in renal cortex tissue. Renal PFK‐1 activity was determined by the coupled‐enzymatic assay and expressed as the OD_340 nm_/min/mg protein. *n* = 7–8. **p < 0.01

To further investigate the possible molecular mechanism by which ablation of TIGAR exacerbates Ang‐II‐induced renal fibrosis and injury, we examined the expression of glycolytic enzymes, hypoxia‐inducible factor‐1 alpha (HIF‐1α), and transforming growth factor beta 1 (TGF‐β1), which play a critical role during the development of renal fibrosis and injury. The expression of HIF‐1α was significantly increased in the TIGAR KO mice after Ang‐II infusion, associated with a remarkable increase in the expression of TGF‐β1, suggesting that Ang‐II induced activation of hypoxic and pro‐fibrotic signaling in the TIGAR KO mice (Figure 5a,b, and E). Knockout of TIGAR resulted in an increase in the expression of PFK‐1, but Ang‐II did not affect the level of PFK‐1 (Figure 5a and d). However, the coupled‐enzyme method revealed that PFK‐1 activity was similarly increased in WT and TIGAR KO mice after Ang‐II infusion (Figure 6). Interestingly, the expression of PFKFB3 was significantly decreased in the WT mice after Ang‐II infusion, whereas it was significantly higher in the TIGAR KO mice (Figure 5a and c).

## 4 DISCUSSION

In the present study, we assessed the effect of ablation of TIGAR in renal function, metabolism, interstitial fibrosis, and glomerular injury during Ang‐II‐induced hypertension. Our results showed that Ang‐II only induced mild interstitial fibrosis and glomerular injury in the WT mice, whereas these pathological modifications were more severe in the TIGAR KO mice, associated with elevated expression of glycolytic enzymes, PFKFB3 and PFK‐1, as well as HIF‐1α and TGF‐β1. These findings suggest that TIGAR is involved in the Ang‐II‐mediated renal metabolic, hypoxic, and fibrotic signaling and pathological alterations.

Ang‐II is a major contributor to the progression of renal fibrosis. Ang II‐induced vasoconstriction, tubular sodium transport, cell growth, cytokine release, and fibrosis are mainly mediated through the Ang‐II receptor type 1 (AT_1_R) (Haase, 2011; Mezzano et al., 2001). Luo and colleagues demonstrated that Ang‐II induced endothelial HIF‐1α gene expression via nuclear factor‐κB‐dependent pathway, which contributes to glomerular injury and promotes hypertensive chronic kidney disease (Luo et al., 2015). A previous study demonstrated that the prolyl‐4‐hydroxylase domain (PHD)–hypoxia‐inducible factor‐1 (HIF‐1) pathway mediated the pro‐fibrotic effects of Ang‐II in rat renal medullary interstitial cells (Wang et al., 2011). Moreover, knockout of endothelial PHD2 suppressed the expression of AT_1_R and prevented Ang‐II‐induced upregulation of HIF‐1α/2α and renal fibrosis and injury (Zhao et al., 2020). These studies demonstrated that hypoxia‐independent HIF signaling is involved in Ang‐II‐mediated fibrosis and glomerular injury. In the present study, the level of HIF‐1α was elevated in the TIGAR KO mice infused with Ang‐II, presumably due to that Ang‐II‐induced ROS generation stabilizes HIF‐1α. TIGAR processes antioxidant effect via increasing the level of NADPH and reducing glutathione (Bensaad et al., 2006) and stimulating hexokinase 2 by translocating to mitochondria under hypoxia (Cheung et al., 2012). Knockout of TIGAR was associated with an increase in ROS (Bensaad et al., 2009), which may further augment Ang‐II‐induced ROS generation and stabilize HIF‐1α. Although our present study revealed that Ang II‐infusion in the WT mice appears to show only mild fibrosis and glomerular injury, knockout of TIGAR significantly sensitizes Ang‐II‐induced renal fibrosis and glomerular injury without altering blood pressure.

HIF‐1α modulates glycolytic flux by up‐regulating many glycolytic genes, including PFKFB3 (Minchenko et al., 2002). Hence, the elevated expression of HIF‐1α and PFKFB3, along with increased PFK‐1 level in the TIGAR KO mice might further increase glycolysis after Ang‐II infusion. Ding et al demonstrated that renal fibroblast activation during renal fibrosis required metabolic reprogramming toward aerobic glycolysis, whereas suppressing renal fibroblast aerobic glycolysis reduced renal fibrosis (Ding et al., 2017). Enhanced glycolytic phenotype has been shown to play a critical role in promoting epithelial–mesenchymal transition (EMT) (Kang et al., 2019). In addition, inhibition of glucose reabsorption and aberrant glycolysis by empagliflozin suppressed EMT and fibrosis in kidney proximal tubules (Li et al., 2020). Knockdown of TIGAR increased glycolysis during severe IRI within 24 h, which is during the early phase (Kim et al., 2015). The increase in glycolysis was beneficial and protected proximal tubular cells from energy depletion and apoptotic cell death. However, Lan and colleagues revealed that the metabolic switch toward glycolysis during the early regeneration phase after acute kidney injury should be reversed for normal recovery, whereas the atrophic tubules persistently exhibited remarkable increase in the expression of glycolytic enzymes associated with high TGF‐β signaling (Lan et al., 2016). Therefore, renal tissue glycolysis should be tightly regulated during insults in a timely manner.

In the present study, knockout of TIGAR increased the expression of TGF‐β1 after Ang‐II infusion in the TIGAR KO mice. TGF‐β1 is induced by Ang‐II and a direct factor that drives fibrosis in CKD. TGF‐β1 promotes renal fibrosis via activation of both canonical and non‐canonical signaling pathways to induce activation of myofibroblasts, EMT, and deposition of excessive of extracellular matrix (ECM) (Kalluri & Neilson, 2003; Kriz et al., 2011; Meng et al., 2016). TGF‐β1 can also stabilize HIF‐1α (McMahon et al., 2006) and induce PFKFB3 gene expression and glycolysis (Rodriguez‐Garcia et al., 2017). In reverse, HIF‐1α mediates TGF‐β1 gene expression (Jiang et al., 2007; Mingyuan et al., 2018), which may result in a feedback loop and promote the formation of renal fibrosis and injury. Taken together, chronic TIGAR deficiency associated increase in glycolysis and pro‐fibrotic signaling during Ang‐II infusion‐induced hypertension contribute to the development of renal interstitial fibrosis. One potential limitation of the present study is that we only measured plasma creatinine levels but did not measure GFR levels. Our data showed that there is a greater glomerular injury score and fibrosis seen in the TIGAR KO mice infusion with Ang‐II while there being no significant changes in plasma creatinine levels. The explanation for this inconsistent is due to a single plasma creatinine measurement maybe not very robust for a 4‐week study. Therefore, an actual measurement of GFR levels is warranted in our future studies.

Collectively, these results demonstrate that TIGAR is important in protecting the kidney from Ang‐II‐induced renal fibrosis and glomerular injury, although it has little effect on blood pressure and renal function. Mechanistically, knockout of TIGAR exacerbates Ang‐II‐induced renal interstitial fibrosis glomerular injury via increasing the expression of glycolytic enzymes, hypoxic, and pro‐fibrotic signaling. This study provides new insights into the role of TIGAR in renal metabolism and pathological remodeling during Ang‐II‐induced hypertension.

## CONFLICT OF INTERESTS

The authors declare that they have no conflict of interest.

## AUTHOR CONTRIBUTIONS

X. He and H. Zeng designed the research; X. He and H. Zeng performed the research and analyzed the data; X. He and H. Zeng wrote the paper. X. He, AC. Cantrell, QA. Williams, JX. Chen, and H. Zeng revised manuscript.

## Supporting information



Table S1

## Data Availability

The data presented in this study are available on request from the corresponding author.
