# Supplementary material for: TIGAR deficiency sensitizes angiotensin‐II‐induced renal fibrosis and glomerular injury
Source: Physiol Rep. 2022 Apr 20;10(8):e15234. doi: 10.14814/phy2.15234 (PMC9020173; doi:10.14814/phy2.15234)
Supplement: Supplementary file 1 — Table S1 [file PHY2-10-e15234-s001.docx]

**Table S1. Antibody list.**

| **Primary Antibodies for Immunoblot Analysis** | | | | | |
| --- | --- | --- | --- | --- | --- |
| **Primary Antibody** | **Catalog #** | **Host** | **Vender** | **MW (kDa)** | **Dilution** |
| β-actin | #86298 | mouse | Cell Signaling, MA | 55 | 1:5000 |
| GAPDH | #2118 | rabbit | Cell Signaling, MA | 37 | 1:5000 |
| HIF-1α | GTX127309 | rabbit | GeneTex, CA | 120 | 1:1000 |
| PFK-1 | sc-377346 | rabbit | Santa Cruz, TX | 85 | 1:1000 |
| PFKFB3 | ab181861 | rabbit | Abcam, MA | 58 | 1:1000 |
| TGF-β1 | sc-146 | rabbit | Santa Cruz, TX | 25 | 1:1000 |
